# Clinical Consequences of Immune Responses to Chlamydia in Men

**DOI:** 10.1155/S1064744996000300

**Published:** 1996

**Authors:** S. Mazzoli, F. Meacci, E. Cosco, C. Poggiali

**Affiliations:** 1 S.T.D. Centre Infectious Disease Unit S.M. Annunziata Hospital U.S.L. 10 H Florence 50111 Italy; 2 Intensive Care Unit Biostatistic Subunit S.M. Annunziata Hospital U.S.L. 10 H Florence Italy


Infectious Diseases in Obstetrics and Gynecology 4:136–142 (1996)
© 1996 Wiley-Liss, Inc.

### Clinical Consequences of Immune Responses to Chlamydia in Men

**S. Mazzoli,[1] F. Meacci,[1] E. Cosco,[1] and C. Poggiali[2]**
[1]*S.T.D. Centre, Infectious Disease Unit, S.M. Annunziata Hospital, U.S.L. 10 H, Florence, Italy*
[2]*Intensive Care Unit, Biostatistic Subunit, S.M. Annunziata Hospital, U.S.L. 10 H, Florence, Italy*

KEY WORDS
*Chlamydia trachomatis* immune response; secretory IgA; IgA1; IgA2; IgA subclasses; prostatitis; male chlamydial infections

*Chlamydia trachomatis* (C.t.) has been involved in a variety of pathological conditions of the female and male genital tracts. Chronic female persistent infections due to this micro-organism have been recently investigated, but the role of C.t. has not been completely clarified especially in acute and chronic pathologies of the male genital tract.

The immunocompetency of the female and male genital tract is well documented[1,2]; the production of secretory IgA (SIgA) can take place in the fallopian tubes,[3] uterine cervix and vagina, prostate and the epididymis, resulting in local production of SIgA by submucosal plasma cells present in a quantity greater than plasma cells producing IgM and IgG. During infections, increased numbers of these three classes of plasma cells are present. Specific SIgA against various infectious agents, such as *Escherichia coli* and *Chlamydia trachomatis*,[4,5,6] are produced in pathological conditions of the genital tract.

SIgA mediates the protection of mucosal membrane by interfering with microbial adherence to mucosal surfaces and by inhibiting the penetration of potentially harmful micro-organisms[7] into mucosal tissues. IgA occurs in serum and secretions as two subclasses that differ in amino acid sequences and glycosylation of the $\alpha$ heavy chain. IgA1 predominates in serum whereas secretions may contain up to 60% IgA2. Subclass composition and response may be important parameters in understanding the host immune response, but the physiological significance of the two subclasses has not been explained. It has been postulated that IgA1 and IgA2 antibodies may play different protective roles: IgA1 is susceptible to bacterial IgA1 proteases; the degrading effect of the IgA1 protease from *N. gonorrhoeae*[8] on anti sperm antibodies suggested a predominant local IgA1 response at least to some antigens but the actual in vivo situation has never been proved. The role of IgA1 proteases has been demonstrated in relation to dental flora: they may promote the adherence of oral streptococci to tooth surface.[7] In 1992, a very strong SIgA protease was demonstrated to be associated with the spermatic fraction of human semen.[9] IgA2 is protease resistant and plays a true role in mucosal defense. The subtle functional differences between IgA1 and IgA2 became recently apparent but, their biological effective role is not clarified.

The appearance of elevated SIgA in seminal plasma, especially during infections, seems to be related to a pathological rather than a physiological situation[2] of the male genital tract. The overproduction of SIgA could be theoretically explained by release of cytokines such as interferon $\gamma$, interleukin (IL) 4 and 5, and tumor necrosis factor (TNF$\alpha$), as demonstrated in the gastrointestinal tract. Otherwise, recently, TNF$\alpha$, and, more recently, other LPS induced cytokines, IL-1 and IL-6, were shown

*Address correspondence to Sandra Mazzoli, P.H.D., Specialist in Microbiology, S.T.D. Centre Coordinator and S.T.D. Laboratory Responsible, S.T.D. Centre, Infectious Disease Unit, S.M. Annunziata Hospital, U.S.L. 10H, Via dell'Antella 58, Antell, 50111 Florence, Italy.

Received September 15, 1996

Article

Accepted October 1, 1996

to be present, in vivo,[10] in mice infected by MoPn *Chlamydia trachomatis* biovar, enhancing a protective role against the infection. TNFα may play a determinant role in other cytokine pathways, leading to enhanced host defence and it is involved in the T-cell independent pathway of macrophage activation. Its production has been recently demonstrated as proportional to the intensity of the infection with high levels coincident with marked neutrophil influx.[11] Cytokines are important mediators of inflammation and their production may modulate immunity during *Chlamydia trachomatis* infections as proved by recent in vitro studies. The role of their induction, especially for IL-1 and IL-6, is actually unclear during several infections.[9] A recent paper[10] concluded that IL1 and IL6 production in vivo in mice could play a role in the pathogenesis and host defence of *Chlamydia trachomatis* infection. Additionally the immune response to Chlamydiae may be immunopathological, as was demonstrated in 1969 in trials of trachoma vaccination in children in Gambia[13] in which the severity of the disease in terms of clinical score was paradoxically greater in vaccinated than in unvaccinated controls. In addition, mice sensitized by immunization with a crude extract or with chlamydial heat shock protein suffered more severe inflammation when challenged intravaginally with live mouse pneumonitis agent organisms.

The cytokine content of patients' biological fluids may be a good indicator of the possible evolution and shift of the immune response, TH1 or TH2, mediated by IFNγ and IL-12 or IL-4. The TH1 → Th2 shift itself may be involved in immunopathological conditions of the genital tract during *Chlamydia* infections, as demonstrated in human immunodeficiency virus pathogenesis studies in vitro.[14,15] Several recent studies suggest that TH 1 cells are crucial in the resolution of established Chlamydial infections.[14,15] Additionally *C. trachomatis* was demonstrated to induce IL-8 secretion in in vitro infected HT29 cells, independent of IL1α stimulation.[16] IFNγ affects the IL-8 induction pathway that does not involve IL1α. This suggests that T-cell-derived cytokines may also control the host response to *Chlamydia* by altering the ability of non-immune cells to respond to the infection, by secreting proinflammatory cytokines and thus increasing the number of inflammatory cells at the site of infection. Cellular immunity is, on the other hand, important in resolving and eradicating established chlamydial infections and pre-existing anti-MOMP antibodies are unable to protect against inital mucosal colonisation (25). In addition IL-10 is the major inhibitor of TH1 responses and of INFγ.

### OBJECTIVE OF THE STUDY

Starting with the previous evidence and the observation of inflammatory symptoms in acute and chronic prostatitis patients, we investigated sera and mucosal secretions for the presence of IL-6 and other cytokines (IL-4, IL-10) in relation to anti-C.t. IgA and IgG and to IgA subclasses in seminal fluids of selected men affected by "abacterial" prostatitis. In addition we studied the concomitant presence of chlamydial DNA by PCR techniques; the global aim of our study was to possibly interrelate all these immunological parameters with the pathological situation and progression to chronicity in our patients.

### MATERIALS AND METHODS

Our study population of 28 patients was selected starting from 290 outpatients admitted to our S.T.D. Centre, Infectious Diseases Unit, S. M. Annunziata Hospital, Florence Italy; the patients were affected by prostatitis or prostatovesciculitis.

The prevalence of anti C.t. specific SIgA in this population was 46.5% (135 positives). The patients' study inclusion criteria were the presence of acute or chronic prostatitis or prostatovesciculitis, clinically and echographically documented, the presence of a strongly positive screening test for species specific anti C.t. IgA in seminal fluids and serum.

Exclusion criteria were the presence of mycoplasmata, bacteria, protozoa, fungi in first morning urine and seminal fluids; negativity for sperm IgA, and antibiotic therapy carried out in the previous six months.

18/28 (64.2%) patients declared symptoms in the past 3 years and 4/28 patients (14.28%) for at least 5 years. All had symptoms since at least six months. Other signs and symptoms were present: prostatic microcalcifications, urethral discharge, stanguria, hematuria, perineal tenesmus, groin, pelvic or ejaculation pains, lymphoadenopathy. Previous S.T.D. infections were mainly H.P.V. infections (10/28). Patients ranged from 26–73 years old with a mean age of 39.1; 85% of patients were between 26–50 years old.

The patients' biological materials that were analysed were sera, semen and urethral swabs. Immediately after sampling, sera were centrifuged and frozen at −40°C, if not immediately tested. To obtain idoneous urethral samples patients were required to return to the laboratory on at least 3 different subsequent days for 3 different samples: slides for Direct Immunofluorescence obtained by endourethral swabs, swabs in the idoneous transport medium for the ELISA test and samples from urethra for Amplicor PCR. All samples were immediately evaluated by the 3 methods. Our diagnostic protocol included a search for C.t. in patients' urethra by DIF (Bio Merieux, France), ELISA (Abbott Diagnostics, USA), and Amplicor *Chlamydia trachomatis* PCR (Roche Diagnostic System, Switzerland), a new sensitive and specific test on this sample.[23]

Semen was aliquoted for the various tests according to the sample amount by the following described procedures and frozen at −40°C until assayed.

PCR on semen was performed by a modified Amplicor PCR. DNA recovery from semen samples was performed by 2 different methods. DNA was extracted from seminal fluids utilising the QIAamp Tissue Kit for rapid purification of DNA for PCR amplification tests. This method was the most efficient procedure for extracting DNA from semen. It provided rapid purification of up to 50 μg of DNA from 200 μl of body fluids. Our modification of the original Amplicor PCR procedure consisted of using for each amplification 50 μl master mix, 50 μl of extracted DNA and 10 μl 25mM $MgCl_2$.

### Cell Culture

Vero cells (African green monkey kidney) were grown in 150 $cm^2$ tissue culture flasks (Falcon) with 199 medium (Biochrom, KG) supplemented with 5% heat inactivated calf fetal serum (Seromed), 0.22% $NaHCO_3$ and 50 μg/ml of gentamicin at 37°C. The cells used in the culture were negative for mycoplasmas. When the cell monolayer was confluent, the growth medium was removed and the monolayer was trypsinized. Cells were suspended in growth medium and counted in a Burker glass chamber. For the assay $2 \times 10^3$ cells per well were seeded into a 24 well tissue culture plate (Falcon) containing cover slips. The culture was incubated at 37°C for 24 hours in a $CO_2$ incubator. The infection of the cells was performed by aspirating the culture medium and replacing it with Eagle's minimum essential medium (MEM) supplemented with 10% fetal calf serum 0.17% $NaHCO_3$, 0.4% glucose, 50 μg/ml gentamicin, 1 μg/ml cyclohexamide (Sigma) containing *Chlamydia trachomatis* serotype LGV2-434 Bu strain at 1–5 inclusion forming units per cell, to insure infection of 80% of the cells. The infected cultures were incubated at 36.5°C for 48 hours in a 5% $CO_2$ thermostat. At this time 50–80% of the cells showed typical cytoplasmic inclusions detectable by phase-contrast inverted microscopy and stains. The medium was then aspirated and the cells washed 2 times with phosphate-buffered saline (PBS). The cover slips were fixed for 10 minutes with methyl alcohol cooled to −10°C, dried and kept at −20°C before use as antigens in indirect immunofluorescence test to detect anti-chlamydial secretory IgA.

Specific anti C.t. immunoglobulins of the G, A classes were detected in semen and sera by Indirect Immune Peroxidase (IIP) test (IPAZyme, Savyon D. Ltd., Israel), Microimmunofluorescence (MIF) test (Labsystem, Finland), ELISA tests (SeroElisa by Savyon D. Ltd., Israel) and Chlamydia rElisa (Medac Diagnostika, Germany). In the present study, we used as antigens to detect specific anti C.t. SIgA both the purified elementary body (MIF) and whole cells infected by this organism: McCoy cells (Savyon) or Vero cells (our original culture) infected by LGV2 C.t. serotype. Secretory component, IgA1 and IgA2 were detected by an original indirect immunofluorescence technique utilising fluorescine isothiocyanate labeled polyclonal antibodies (Dakopatts, Denmark; The Binding Site Ltd. U.K.). Biological material under study were absorbed with the various antigenic substrates for 30 minutes at 37°C in a humid chamber; after washing in PBS for at least 10 minutes, the fluorescine conjugate was absorbed for 30 minutes at 37°C. Testing dilutions of the conjugates were made in accordance with the manufacturers' recommendation. Negative and positive controls were run with each reaction. A final washing step was performed in PBS and observation was immediately done under fluorescence microscopy at 40× magnification: secretory component showed a fine diffuse fluorescence positivity which was confirmed in immersion fluorescence microscopy at 100× magnification.

### Interleukin Assays

IL-6 was detected by a quantitative ELISA method (Eurogenetics, Belgium) utilising anti-IL6 monoclonal antibodies. Blood samples were collected in Vacutainer B.D. glass tubes and left to sediment 1 hour at room temperature before centrifuging the samples (1700 g., 30 min., 4°C). We collected the upper two-thirds of the supernatants (1 ml) and stored at −40°C until assayed. Serum samples were aliquoted and one aliquot was decomplemented (20 min. 56°C.) before testing to destroy the possible interfering action of the soluble IL-6 receptor, which binds IL-6 in solution. Bioassays of sera were performed in duplicate to verify if increments of the concentration could be present in the decomplemented fraction. Quantification of the IL-6 content was performed by using five standards of 10, 25, 50, 200 and 500 pg/ml IL-6. The standards were calibrated against the "Unclassified Interleukin-6 (recDNA human type) 88/514". One vial containing 1 ng lyophilised IL-6 was used as a recovery control positive test and was reconstituted with a negative semen. Normal values in our population were previously calculated for semen starting from a selected normal population negative for signs and symptoms of prostatitis and for all bacterial, mycoplasmal and other microbial infections. IL-4, IL-10 were detected by quantitative ELISA tests (Biokine, T-cell Diagn. Inc. and Predicta Genzyme, USA).

### Statistical Methods

The Wilcoxon test for paired data was used to determine the comparison between the detected values of all our immunological parameters by Labstat version 3.03 program for Biostatistic. MS DOS SyStat program and SysGraph for Window were used for statistical analysis and graphics.

### RESULTS AND DISCUSSION

Specific anti *Chlamydia trachomatis* IgA was a constant component in patients' seminal fluids by all our methods of detection; both of the ELISA tests detected a very high content of IgA with 75% agreement for absorbance values over 2.000. Statistical analysis on IgA means (SeroELISA test and Medac r-ELISA) obtained in semen and sera, revealed statistically significant differences ($p = 0.000$), reflecting local overproduction of IgA in semen and, thus, in tissues.

Higher levels of anti LPS IgA detected in semen was in accord with animal studies in which the persistence and recovery of high levels of IgA in secretions of the genital tract seem to be related to a booster[16] effect only at the site of infection. Thus, in prostatitis patients this could be related to the persistence of the infection in the male upper genital tract.

The recombinant anti-LPS test showed the presence of a higher level of anti-LPS specific anti C.t. antibodies of both the two classes: IgA and IgG. Semen IgG against C.t. LPS were demonstrated in 12 patients by rELISA as compared with 5 patients by SeroElisa; IgG detection in seminal fluids was important to verify if the local immunisation in these patients was locally produced or a translocated extension of humoral immunity, transudated from sera. IgA1, IgA2 subclasses were detected in seminal fluids and sera of all 28 patients except 2: IgA1 was demonstrated in semen of 8/28 patients (28.5%). 93.7% of the patients declaring symptoms in the previous 3 years were positive for IgA2, making IgA2 immune response strictly related to the persistence of the infections. IgA2 was mainly present in our patients (92.8%) confirming, as in our previous observations, their presence as the most-expressed and significant immunoglobulin subclass in semen. These data seem to be in agreement with the production of IgA1 proteases in this biologic fluid. Otherwise, the presence of IgA1, in the majority of the semen samples containing detectable levels of IgG, seems to correlate well with the possible transmembrane translocation or transudation of serum IgG, probably due to permeability damages of the blood-genital tract barrier, caused by inflammation and its powerful mediators.

IL-4 was also assayed but this lymphokine was not detectable. IL-10 was present in high levels in 75% (25/28) of our patients and very high values were present in IgA2 positive patients.

IL-6 was present at a fourfold higher concentration (>20 pg/ml) than normal serum levels (5 pg/ml) in 75% (21/28) of our selected patients seminal fluids. Comparison of IL-6 detected in sera and semen showed a statistically significant difference ($p = 0.001$) between mean concentrations, in favour of a local overproduction of this cytokine. If we plot IL-6 (semen) vs. IgG and IgA detected (Savyon SeroElisa.) in semen of our patients we obtain Figure 1; IL-6 values were distributed in three groups on the IgG axis. The majority of our IgG detection coincided with no IL-6 or with very low values, a smaller group in proximity of the cut-off and sparse observations on higher values. Observing the distri-

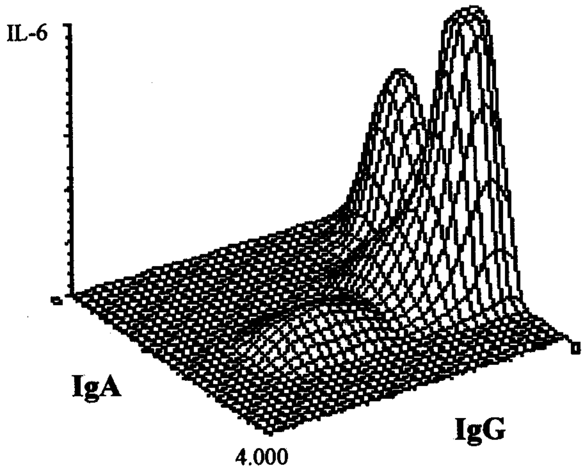

Fig. 1. In asciss axes the Optical Densities (O.D.) of the IgG and IgA (from 0 up to fictitious value 4.000; real values detected till 3.000) detected in semen of our patients by SeroELISA test; in ordinate axis IL-6 O.D. It is possible to distinguish three different subpopulations: the first one with zero values of IgG and medium values of IgA, the second one with very high values of IgA and zero values of IgG and the third one with very high values of IgA and high values of IgG; included in the last one are the IgA1 positive patients, showing contemporary presence of IgG and IgA1 subclass antibodies probably of serum origin through a transmembrane translocation process.

bution from the IgA axis' point of view, we have a first group aligned on the 3.000 optical density value (fictitious value attributed to values read as "Over", by ELISA test). This group contains also the higher values of IL-6, roughly as far as 2100 value for IgG. A second group defines patients under O.D. 2.000 and lower.

Secretory component of the IgA was investigated by using different antigens. The presence of 3 negative patients utilising purified EB. of C.t. LGV2 serovar, may be explained with the possibility of the lack of 20% specific immunization utilising one single *C.t.* serotype, or, more likely, with the presence of polymeric IgA against different antigens, or with the presence of monomeric IgA in seminal fluids.

Otherwise, 89.2% of these patients had evidence of secretory component. Thus the IgA immune response was a secretory related response. Mucosal surfaces in direct contact with the external environment, including the genital tract, have a common immune system with selective traffic of B and T lymphocytes from GALT and BALT.[16] The specific anti C.t. response in our patients is independent of the response in serum and mostly associated with secretory IgA.

It was not possible to interrelate chlamydial DNA positivity with other markers of infection, because of the negative PCR results obtained in seminal fluids; we can only affirm that 28.5% (8/28) of our patients were PCR positive for C.t.DNA, confirming the presence of the micro-organism as a possible "primum movens" of the pathology and its presence. Another reason for negative PCR results could be the presence of very high values of SIgA: our finding seems to confirm animal model findings[17] in which an inverse correlation between specific SIgA titre in genital secretion and the presence of *Chlamydia trachomatis* isolated by culture in the cervix was found.

### CONCLUSIONS

We conclude that our patients with anti *Chlamydia trachomatis* IgA positive "abacterial" prostatitis have "Chlamydial" prostatitis; they presented with specific local immunisation against this microorganism, a local overproduction of IgA confirming the proper immunocompetency of the genital tract, independent from the systemic humoral one, probably not protective (prevalence of DNA detection at a high rate) and continuously boostered by antigen elimination. Induction of IgA2 seems to be strictly related to the persistence of symptoms and, thus, of infection. Moreover, IgA2 is related to the type of antigenic stimulation[18] at the mucosal site and to repeated antigenic stimulation, more than to a biological shift in local IgA production, caused by destruction of IgA1 by bacterial proteases. This hypothesis seems to find confirmatory reports in a recent paper of Kawamura and co-workers,[19] demonstrating that the ancestral $\alpha$ chain genes are closely related to the $\alpha$ 2 and not $\alpha$ 1 ones of humans and hominoid primates. Future studies will be extremely useful in expanding our understanding of the biological role of these antibodies.

The presence of IgA2 in the case of chronic prostatitis in our population is a confirmatory marker of the persistence and pathology of the chlamydial infection. Secretory IgA themselves, in semen of our chronic prostatitis patients (symptoms since 3–5 years to at least 6 months), is an index of "active" infection and allows us to detect and to define "chronic active infections" of the prostate.

Our patients presenting with chronic prostatitis were very rich in symptoms, mainly those with corresponding high levels of secretory IgA in seminal fluids, confirming that anti C.t. IgA total content and SIgA concentration is a reliable index of infectious activity. These reports are, in addition, emphasized by the contemporary association of elevated values of IL-6, whose importance in inflammatory phase processes is well known.[20]

The local production of IL-6 is well documented in our patients affected by prostatitis. Thus, we can confirm the production of this multifunctional cytokine in human chlamydial infection in vivo. IL-6 is strictly correlated to the anti *Chlamydia trachomatis* secretory and total IgA content in seminal fluids and doesn't seem to affect this immunoglobulin production, if not as a stimulatory factor. Like in the mouse[21] IL-6 production may reflect the $CD4^+$ T cells helper function in genital tract mucosae by providing help to mucosal B cells for the production of *Chlamydia*-specific IgA. IL-4 was not detectable and this fact may be due to its rapid turnover in body fluids or to the fact we can detect only terminal TH2 cytokines. The recovery of IL-10 in patients with chronic symptoms and IgA2 was extremely interesting. IL-10 is the major inhibitor of IFN$\gamma$ which induces inhibition of Chlamydial growth in vitro[22,23,24] and resolution of Chlamydial infections.[25] Thus the presence of IL-10 in our chronic patients confirms that, in these patients too, cell mediated immunity is depleted and the TH2 immune shift seems correlated with the persistence of the infection and with the overproduction of IgA antibodies and with the adverse pathological events in the target organ, the prostate gland.

The biological role of IL-6, other cytokines and secretory IgA overproduction in modulating the passage from acute to chronic prostatic infection and micro-organism clearance, is emphasized by our findings, and the possibility that the pathogenesis of Chlamydial prostatitis could be immune complex related has to be thoroughly investigated in the future.

### REFERENCES

1. Mestecky J.: The common mucosal immune system and current strategies for induction of immune responses in external secretions. J Clin Immunol 7:265–276, 1987.
2. Brandtzaeg P, Purvis K.: Immunological investigations of the genital tract. Uppsala J Med Sci Supplement 50:62–65, 1991.
3. Kutteh W, Blackwell R, Gore H, Kutteh C, Carr B, Mestecky J.: Secretory immune system of the female reproductive tract. II. Local immune system in normal and fallopian tube. Fertil Steril 54:51–55, 1990.
4. Tsunekawa T, Kumamoto Y: A study of IgA and IgG titers of *C. trachomatis* in serum and prostatic secretion in chronic prostatitis. J Infect Dis 63:130–137, 1989.
5. Mazzoli S, Salis S: Detection of IgA specific antibodies anti Chlamydia trachomatis STDs serotypes in secretions: Clinical significance and patients follow-up after therapy. EOS:IX, 3, 181, 1989.
6. Mazzoli S, Spina C, Piccinin L, Benaim G: Specific anti-Chlamydia immune response and IgA subclasses composition in male patients affected by infertility and prostatitis (abstract). Eur Soc Inf Dis Obstet Gynecol, Taormina June 1992.
7. Ahl T, Reinholdt J: Subclasses distribution of salivary secretory immunoglobulin A antibodies to oral Streptococci. Infect Immunol 59:3619–3625, 1991.
8. Mulks MH, Knapp JS: Immunoglobulin A1 protease types of *Neisseria gonorrhoeae* and their relationship to auxotype and serovar. Infect Immun 55:931–936, 1987.
9. Olivares Morales S, Vadeillo Ortega F, Villanueva Diaz C, Hernandez Guerrero C, Arredondo Garcia J: Identification and initial characterisation of a protease of immunoglobulin A associated with human spermatozoa. Ginecol Obstet Mex 60:67–74, 1992.
10. Magge DM, Smith JG, Bleicker CA, Carter CJ, Bonewald LF, Schachter J and Williams DM: *Chlamydia trachomatis* pneumonia induces in vivo production of Interleukin-1 and -6. Infect Immunol 60:1217–1220, 1992.
11. Darville T, Laffoon KK, Kishen LR, Rank RG: Tumor necrosis factor alpha activity in genital tract secretions of guinea pigs infected with chlamydiae. Infect Immun 63:4675–4681, 1995.
12. Hedaes S, Srenavist K, Lidin-Janson G, Martinell J, Sandberg T, Svanborg C: Comparison of urine and serum concentrations of interleukin-6 in women with acute pyelonephritis or asymptomatic bacteriura. J Infect Dis 166:653–656, 1992.
13. Sowa S, Sowa J, Collier LH, Blyth WA: Trachoma vaccine field trials in The Gambia. J Hyg 67:699–717, 1969.
14. Clerici M, Shearer GM: Is HIV associated with TH1 → TH2 switch? Immunol Today, 14:107–111, 1993.
15. Clerici M, Shearer GM: The TH1/TH2 model of HIV infection: New insights. Immunol Today 15:575–581, 1994.
16. Cui ZD, Tristram D, La Scolea LJ, Kwiatkowski T Jr, Kopti S, Ogra Pl: Induction of antibody response to *Chlamydia trachomatis* in the genital tract by oral immunisation. Infect Immunol. 59:1465–1469, 1991.
17. Brunham RC, Kuo CC, Cles L, Holmes KK: Correlation of host immune response with quantitative recovery of *Chlamydia trachomatis* from the human endocervix. Infect Immunol 39:1491–1494, 1983.
18. Miettinem A, Heinonen PK, Teisala K, Punnonen R,

Paavonen J: Antigen specific serum antibody response to *Chlamydia trachomatis.* J Clin Pathol 43:758–61, 1990.

19. Kawamura S, Omoto K, Ueda S: Evolutionary hypervariability in the hinge region of the immunoglobulin alpha gene. J Mol Biol 215:201–206, 1990.
20. Heinrich PC, Castell JV, Andus T: Interleukin-6 and the acute phase response. Biochem J 265:621–636, 1990.
21. Su H, Caldwell HD: CD4+ T cells play a significant role in adoptive immunity to *Chlamydia trachomatis* infection of the mouse genital tract. Infect Immunol 63:3302–3308, 1995.
22. Byrne GI, Carlin JM, Merkert TP, Arter DL: Long term effects of gamma interferon on Chlamydia-infected cells: Microbiocidal activity follows microbistasis. Infect Immunol 57:1318–1320, 1989.
23. de la Maza LM, Peterson EM, Goebel JM, Fennie CW, Czarniecki CW: Interferon-induced inhibition of *Chlamydia trachomatis:* Dissociation from antiviral and antiproliferative effects. Infect Immunol 47:719–722, 1985
24. Shemer Y, Sarov I: Inhibition of growth of *Chlamydia trachomatis* by human gamma interferon. Infect Immunol 48:592–596, 1985.
25. Ward ME: An update on the immunology of Chlamydial infection. Proceedings of the third meeting of the European Society for Chlamydia Research, Vienna, September, 1996.
